# Inferior Wall Myocardial Infarction Complicated With Acute Mitral Regurgitation Requiring Transcatheter Edge-to-Edge Repair

**DOI:** 10.7759/cureus.74861

**Published:** 2024-11-30

**Authors:** Rajendra P Shah, Olayiwola Bolaji, Anderson C Ariaga, Aimen Shafiq, Yasemin Bahar, Timir Paul, Wael AlJaroudi, Rajeev L Narayan, M. Chadi Alraies

**Affiliations:** 1 Internal Medicine, Vassar Brothers Medical Center, Poughkeepsie, USA; 2 Internal Medicine, University of Maryland Capital Regional Medical Center, Largo, USA; 3 Internal Medicine, Dow University of Health Sciences, Karachi, PAK; 4 Internal Medicine, Wayne State University, Detroit, USA; 5 Section of Interventional Cardiology, University of Tennessee at Nashville/Ascension Saint Thomas Hospital, Nashville, USA; 6 Cardiovascular Medicine, Medical College of Georgia, Augusta University, Augusta, USA; 7 Cardiology, Vassar Brothers Medical Center, Poughkeepsie, USA; 8 Cardiology, Wayne State University Detroit Medical Center, Detroit, USA

**Keywords:** left heart catheterization, mitral regurgitation, st-elevation myocardial infarction, transcatheter edge-to-edge repair, transesophageal echocardiography

## Abstract

Acute ST-elevation myocardial infarction (STEMI) is a life-threatening condition often associated with significant cardiac complications, particularly in the presence of underlying multivessel coronary artery disease. Mechanical complications, such as acute mitral regurgitation (MR), can worsen the clinical course, leading to rapid hemodynamic deterioration. Recent advancements in mechanical circulatory support and percutaneous interventions have introduced new therapeutic options, offering viable alternatives to traditional surgery for high-risk patients. In this case, a 67-year-old male with a history of hypertension presented with sudden-onset chest pain and dyspnea. Electrocardiography revealed an inferior STEMI. En route to the catheterization laboratory, the patient experienced cardiac arrest and was subsequently found to have multivessel coronary artery disease and severe mitral regurgitation. The patient underwent Impella-assisted multivessel percutaneous coronary intervention, complicated by flash pulmonary edema, which necessitated transcatheter mitral valve repair. The patient remained hemodynamically stable and had a successful recovery post-intervention. This case shows the effective use of advanced percutaneous techniques and mechanical support in managing a complex cardiac emergency, highlighting their potential as alternatives to traditional surgery.

## Introduction

ST-elevation myocardial infarction (STEMI) is sometimes associated with mechanical complications such as acute mitral regurgitation (MR), ventricular septal rupture, pseudoaneurysm, and free wall rupture, which tend to cause worse outcomes and prognosis. Risk factors for these complications include older age, comorbidities, and delayed reperfusion [1]. Acute MR usually occurs around three to five days following a STEMI and can lead to acute pulmonary edema and shock. It is most associated with inferior or lateral STEMIs involving the right coronary artery or circumflex artery territory. The mitral valve comprises two cusps held by the posteromedial and anterolateral papillary muscles. The anterolateral branch has a dual arterial blood supply from the left anterior descending artery (LAD), and either the diagonal artery or marginal branch of the circumflex coronary artery, the posteromedial papillary muscle has a single blood supply from either the circumflex coronary artery or the right coronary artery (RCA), depending on dominance. This singular vessel supply makes the posteromedial muscle more susceptible to ischemic and subsequent rupture. The current standard of care includes stabilization of the patient with temporary circulatory support such as Impella or Intra-aortic balloon pump (IABP) with surgical repair with or without coronary artery bypass grafting (CABG) if there is severe coronary artery disease [1,2]. This case is noteworthy due to the use of the Impella device in a patient with inferior STEMI and subsequent acute ischemic MR. The patient was managed with Impella support and underwent high-risk percutaneous coronary intervention (PCI) and eventually had a MitraClip procedure as compared to surgical valve replacement without any complications.

## Case presentation

**Figure 1:**
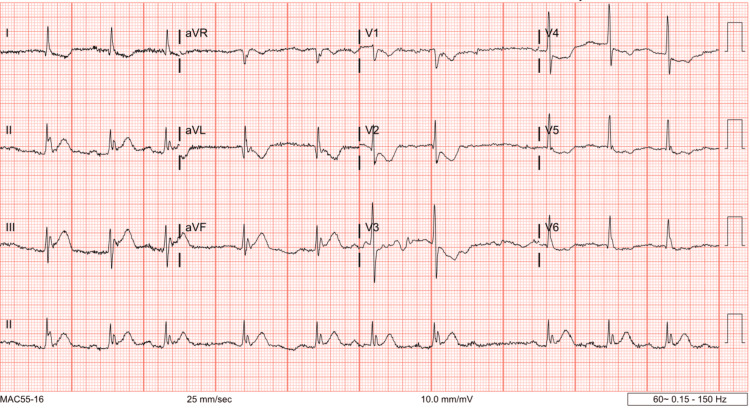
EKG showing ST elevation myocardial infarction

A 67-year-old man with a past medical history significant for hypertension presented to Vassar Brothers Medical Center, Poughkeepsie, with sudden onset chest pain and dyspnea. EKG showed inferior wall STEMI (Figure 1).

En route to the catheterization lab, the patient had a cardiac arrest with ventricular fibrillation and cardiogenic shock. After successful resuscitation, emergent left heart catheterization and coronary angiogram showed a critical lesion in LAD, Circumflex, and RCA territories (Videos 1-3). Transesophageal echocardiography (TEE) demonstrated severe MR with P2 flail segment with concern for acute ischemic MR. Cardiothoracic surgery consultation was obtained, and the patient was found to be of prohibitive risk for emergent CABG. As a result, Impella-assisted multivessel PCI was performed, and the patient was initially liberated from mechanical ventilation. The patient, however, was reintubated due to flash pulmonary edema, and a follow-up TEE revealed and confirmed severe MR. The heart team's decision was made for transcatheter mitral valve repair. The patient underwent successful implantation of the XTW mitral clip G4 system with a reduced MR. The patient remained hemodynamically stable. The patient had a successful postoperative period and was eventually liberated from mechanical ventilation and uneventful outpatient follow-up. The patient was discharged from the hospital and is doing better.

**Video 1:** Coronary angiography showing left circumflex lesion

**Video 2:** Coronary angiography showing left main and left anterior descending lesion

**Video 3:** Coronary angiography showing right coronary lesion

## Discussion

Acute severe MR is one of the known complications of STEMI. Severe MR will need valve repair at the time of stenting to improve survival [2]. Acute management includes mitral valve repair and supportive therapies [3]. Ultimately, this case demonstrated the rare use of the Mitraclip in acute STEMI. The successful management of this case underscores the potential of advanced percutaneous techniques and mechanical support devices like Impella in treating complex STEMI-related complications. The Impella device provided crucial hemodynamic support, allowing for high-risk PCI and facilitating the MitraClip procedure [4]. This approach presents a viable alternative to surgical valve replacement, particularly for patients with significant comorbidities or those at high surgical risk. The favorable outcome in this patient suggests that a multidisciplinary approach incorporating mechanical support, advanced PCI, and percutaneous valve interventions can be highly effective, reducing the need for more invasive surgical procedures.

## Conclusions

This case presents a novel approach to managing acute mitral regurgitation in STEMI with cardiogenic shock, using Impella-assisted PCI followed by MitraClip. The positive outcome underscores the potential of advanced percutaneous techniques and mechanical support in treating complex cardiac emergencies, offering a less invasive alternative for high-risk patients. While promising, this case highlights the need for further research to confirm broader applicability and long-term outcomes. Careful patient selection and specialized cardiac care are essential. As interventional cardiology advances, implications for medical training and heart team protocols should be considered.
